# Correlation between Psoas Muscle Index and Degeneration of Spinal Back Muscle in Patients with Back Pain

**DOI:** 10.3390/healthcare9091189

**Published:** 2021-09-09

**Authors:** Donggyu Lee, Minsoo Kang

**Affiliations:** Department of Physical Medicine and Rehabilitation, College of Medicine, Yeungnam University, Daegu 42415, Korea; painfree@yu.ac.kr

**Keywords:** spine sarcopenia, aging, psoas muscle index, chronic low back pain, multifidus muscle

## Abstract

Sarcopenia is characterized by a decline in systemic muscle mass and physical performance. Disc degeneration also causes back muscle atrophy. Therefore, we aimed to evaluate the influence of systemic muscle mass decline on back muscle atrophy and fatty infiltration compared to disc degeneration. We included 127 patients (65.54 ± 14.93 years) with back pain who underwent lumbar spine magnetic resonance imaging (MRI). Axial T2-weighted MRI data of the L4–5 and L5-S1 levels were used to measure the cross-sectional area (CSA) of the psoas and spinal muscles. The psoas index (cm^2^/m^2^) was used as a surrogate for systemic muscle mass. The Pfirrmann grading system was used to evaluate intervertebral disc degeneration. The functional area of the back muscles was calculated by subtracting the fat infiltration area from the CSA; the functional CSA ratio was calculated by dividing the functional CSA by the CSA. Image-processing software (ImageJ; National Institutes of Health, Bethesda, MD, USA) was used for analysis. Psoas index and aging significantly affected CSA and the ratio of functional CSA of the back muscles and multifidi. Disc degeneration did not significantly affect the back muscles beyond aging in patients with back pain. Males showed substantially higher CSA of the back muscles and multifidi than females; however, sex did not affect the functional CSA ratio of these muscles. Systemic muscle mass decline showed a more powerful influence on back muscle atrophy and fatty infiltration than disc degeneration. Therefore, proper evaluation of sarcopenia is needed for patients with chronic back pain and back muscle degeneration.

## 1. Introduction

Core stability is the principal factor in spinal stability and skeletal muscle activation. The muscles involved in core stability are activated before those of the extremities. Moreover, these muscles support the activities of the extremities [1]. The back muscles play a role in core stability. Disc degeneration was positively correlated with multifidus atrophy or fatty infiltration [2]. Furthermore, disc degeneration and spinal stenosis can injure the spinal nerves, which innervate the multifidi [3]. Consequently, patients with back pain show paraspinal muscle atrophy [4,5]. Activation patterns in the back and extremity muscles change in back pain patients [1]. Based on this notion, researchers have regarded back muscles as a key factor in chronic back pain. Core muscle strength training has been the focus of exercise treatment for patients with back pain. However, conflicting data reported that the cross-sectional area (CSA) of the back muscles was not significantly different between patients with LBP and healthy controls [5]. Sarcopenia is defined as low skeletal muscle mass and reduced muscle strength and physical performance in old age [6]. Sarcopenia and back muscle atrophy are closely associated with regard to general muscle atrophy. Moreover, most disc pathologies result from degenerative changes associated with aging. Accordingly, back muscle atrophy or fatty infiltration can also be a component of sarcopenia, similar to appendicular skeletal muscle atrophy.

There are many tools and patterns available for the measurement of appendicular skeletal muscle mass (ASM) or CSA of specific muscle groups [6]. Dual-energy X-ray absorptiometry (DXA) and bioelectrical impedance analyses are some examples. Alternative measurement methods have also been used to determine muscle quantity. The lumbar L3 CSA, mid-thigh muscle, and psoas muscle index can be used as surrogates for ASM. Although there are limitations of single muscle measurement in the assessment of muscle quantity, it is a simple and convenient method to measure increased usage in sarcopenia studies [7]. The psoas is located around the lumbar spine and innervated by the upper part of the L1-L3 spinal nerves. Thus, we used the psoas index (psoas muscle CSA/height^2^) as a surrogate for ASM in this study.

In this study, we aimed to determine the components of disc degeneration and sarcopenia. Loss of systemic muscle mass was hypothesized to be associated with back muscle atrophy and fatty infiltration in patients with chronic back pain.

## 2. Materials and Methods

### 2.1. Subjects

We performed a retrospective study of patients who visited our university hospital spine center for low back and/or radicular pain from 2018 to 2021. This study was approved by the institutional review board (YUMC 2021–07-042). The requirement for informed consent was waived. Patients aged ≥20 years who underwent lumbar spine magnetic resonance imaging (MRI) were included in the study. The exclusion criteria were as follows: (1) absence of height and body weight information, (2) history of spinal surgery, (3) history of spinal infection, (4) scoliosis, (5) history of spinal cord injury and subsequent inability to walk independently, and (6) history of cerebrovascular disease.

### 2.2. Measurements

**Figure 1:**
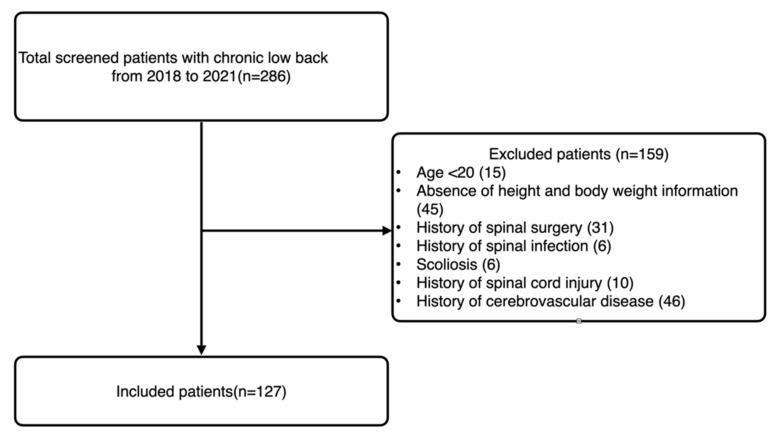
Flowchart of patient inclusion and exclusion.

All included patients underwent spinal MRI (1.5T MRI scanner, Siemens, Germany). A total of 127 patients with back pain were included (Figure 1). The mean age was 65.54 ± 14.93 years. Sagittal T2-weighted MRI was used to evaluate the disc degeneration. Axial T2-weighted MRI (repetition time/echo time, 3400/111; field of view, 157 × 180; and matrix size, 384 × 235) was used to measure the CSA of the psoas and spinal muscles. Fatty infiltration in the back muscles was also evaluated based on the axial T2-weighted images.

### 2.3. Pfirrmann Grading System

The Pfirrmann grading system was used to evaluate intervertebral disc (IVD) degeneration at the L4–5 and L5–S1 IVD levels [8]. This system is based on the disc signal intensity, the distinction between the annulus and nucleus, and the disc height. The grading system is defined as follows: Grade I: the structure of the disc is homogeneous, with a bright hyperintense white signal intensity and a normal disc height; Grade II: the structure of the disc is inhomogeneous but maintains a hyperintense white signal, and a horizontal margin may be present. The nucleus and annulus have distinct margins, and the disc height is normal; Grade III: the disc is inhomogeneous with intermediate gray signal intensity. The distinction between the nucleus and annulus is unclear, and the disc height is normal or slightly decreased; Grade IV: The structure of the disc is inhomogeneous with a hypointense dark gray signal intensity. There is no distinction between the nucleus and annulus, and the disc height is normal or moderately decreased; Grade V: the disc is inhomogeneous with hypointense black signal intensity. There is no distinction between the nucleus and annulus, and the disc space is collapsed.

### 2.4. CSA and Fatty Infiltration Measurement

**Figure 2:**
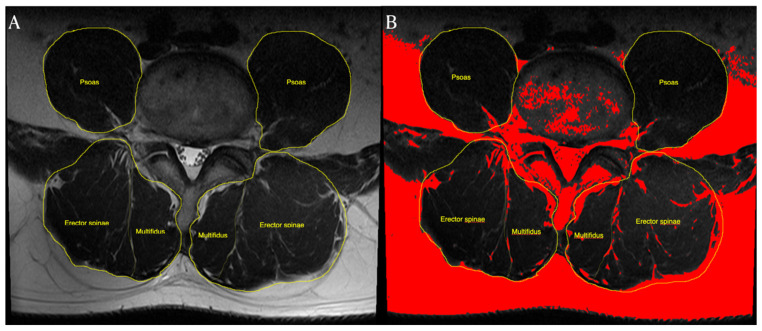
We defined the back muscles as consisting of the multifidi and erector spinae muscles. This region was manually traced. The CSA of the back muscles and multifidi were measured separately: (**A**) the rate of fat infiltration was measured using the pseudocoloring technique in the software. Fat tissue on axial T2-weighted MR image was converted to red. We calculated the portion of red area among the CSA of back muscles (**B**).

CSA measurement was performed using axial T2-weighted images at the L4–5, and L5–S1 levels. We used image-processing software (Image J; National Institutes of Health, Bethesda, MD, USA) to measure the CSA and fatty infiltration of muscles. DICOM files from the MRI were mounted using the Image J program. Subsequently, CSA measurements were performed by manually tracing the boundaries of each muscle. The CSA of the psoas muscle was measured at the L4–5 level. The psoas index (cm^2^/m^2^) was calculated (psoas index: bilateral psoas muscle CSA/height^2^) to evaluate potential sarcopenia [9]. In this study, we defined the back muscles as consisting of the multifidi and erector spinae muscles. This region was manually traced by following the thoracolumbar fascia, lateral margin of the spinous processes, posterior facets, cortical margin of the laminae, and dorsal side of the quadratus lumborum. The CSAs of the erector spinae (back muscles) and multifidi were measured separately (Figure 2A).

The rate of fat infiltration was measured using a pseudocoloring technique. We converted the 16-bit image to a pseudocolor 8-bit image. We then adjusted the threshold to assess fat infiltration. The fat tissue on the axial T2-weighted MR images was converted to a red color. We calculated the portion of the red area among the CSAs of the back muscles (Figure 2B). The functional area of the muscle was calculated by subtracting the fat infiltration area from the CSA of the back muscles [10]. The ratio of functional CSA of the back muscles and multifidi was calculated by dividing the functional CSA by the CSA.

### 2.5. Statistical Analysis

Statistical analysis was performed using SPSS (version 22.0; SPSS Inc., Chicago, IL, USA), and continuous variables are presented as mean ± standard deviation. A standard *t*-test was used to estimate differences between the sexes. Multivariate regression analysis was used to estimate the factors that significantly influenced the CSA and functional CSA of the back muscle and multifidus. Statistical significance was set at *p* < 0.05.

## 3. Results

The demographic data are summarized in Table 1. Males showed significantly higher CSA and ratio of functional CSA at the L4–5 and L5–S1 levels than females. Moreover, males had a significantly higher psoas index. Hence, male subjects showed preserved functional muscle mass, compared to female subjects. The back muscles at the L4–5 level showed a larger CSA than those at the L5–S1 level, whereas the multifidus at the L5–S1 level showed a larger CSA than that at the L4–5 level.

**Figure 3:**
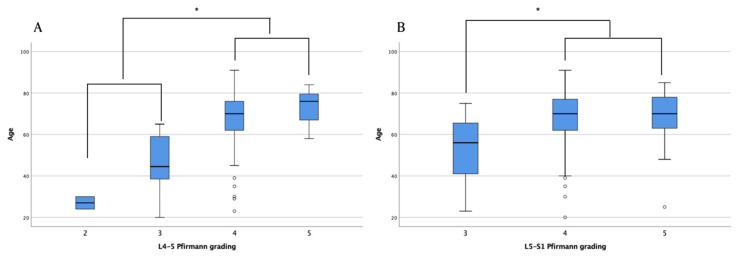
Distribution of age according to the Pfirrmann grading system. Pfirrmann grades 4 and 5 had a higher age than Pfirrmann grades 2 and 3 at L4–5 level (**A**), and Pfirrmann grade 3 at L5–S1 level (**B**), respectively. Older ages showed higher disc degeneration grading. Box and whisker plot present the median (middle line), the 25th and 75th percentiles (box limits). The circles are below 5th percentile of the distribution. * *p* < 0.05.

An association between the psoas index, sex, and CSA was found (Table 2). However, the Pfirrmann classification did not significantly affect the CSA of the back muscles, except for the multifidus at the L5–S1 level. Grades 4 and 5 Pfirrmann classifications were higher in older age groups than in relatively younger age groups (Figure 3). Although the Pfirrmann classification was shown to be associated with age, it did not have a significant relationship with CSA or the ratio of functional CSA (Table 3).

**Figure 4:**
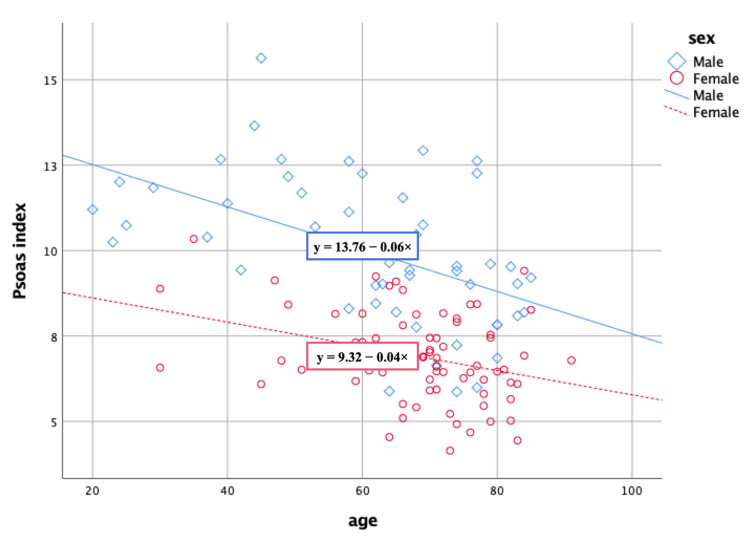
Psoas index and age showing a negative correlation. Age and sex affected psoas index.

The Psoas index was found to be associated with the CSA and ratio of functional CSA of the back muscles and multifidi at the L4–5 and L5–S1 levels. Furthermore, the Psoas index decreased with increasing age (Figure 4). Generalized muscle atrophy and the psoas index were closely associated with back muscle atrophy. Male subjects showed significantly higher CSAs of the back muscles and multifidi than female subjects. However, sex did not affect the functional CSA ratio of these muscles.

## 4. Discussion

Disc degeneration affects back muscle atrophy. However, in our study, the psoas index and aging significantly influenced the CSA and ratio of functional CSA of the back muscles and multifidi, and disc degeneration did not significantly affect the back muscles beyond aging in patients with back pain. These results showed that disc degeneration and generalized muscle atrophy significantly affected back muscle atrophy and fatty infiltration.

Disc degeneration increases the possibility of injury to nerves innervating the back muscles. Moreover, disc herniation produces inflammation that damages the nerve roots, resulting in the denervation of the back muscles [11,12]. Furthermore, in addition to ligament and bony hypertrophy, decreased disc height following disc degeneration causes foraminal and central stenosis [13]. Spinal stenosis also damages nerves innervating the back muscles [14]. Thus, disc degeneration is known to be closely related to injury of spinal nerves innervating the back muscles.

Studies have investigated the relationship between disc degeneration and back muscle atrophy based on the notion that disc degeneration may induce back muscle functional deterioration. Disc degeneration was correlated with multifidus atrophy [8]; however, the relationship was weak. In the previous study, the mean age was 47.22 ± 15.08 years. Moreover, the sample size of 35 patients was small. The younger age and small sample size could have limited the investigation of the influence of age on back muscle atrophy. Disc herniation increased fat infiltration on the ipsilateral side compared to that on the contralateral side [15]. Moreover, ipsilateral injury of the spinal nerve increased fatty infiltration of the ipsilateral multifidus. However, we did not compare both sides of the back muscles and multifidi. Although we did not investigate the asymmetry of back muscles, the clinical significance of this study was the comparison of back muscle atrophy and fatty infiltration with aging and disc degeneration.

Sarcopenia is defined as the loss of generalized skeletal muscle mass and function. Evaluation tools for sarcopenia measured muscle strength and quality, as well as physical performance [6]. Tool selection depends on the technical equipment, the purpose of evaluation, and patients’ clinical situations. Our subjects presented with back pain; therefore, we used MRI to evaluate sarcopenia. The psoas index correlated with skeletal muscle index and skeletal muscle mass adjusted for individual patient heights [16]. Moreover, its cut-off value for sarcopenia correlated well with the skeletal muscle index [17]. Therefore, we used the psoas index for sarcopenia to evaluate the relationship between back muscle atrophy and fatty infiltration.

Various clinical factors, including obesity, diabetes mellitus, myopathy, and peripheral neuropathy, yield intramuscular adipose tissue infiltration [18,19]. Fatty infiltration has also been shown to decrease physical function. Moreover, a decrease in skeletal muscle mass and an increase in intramuscular adipose tissue were found to be associated with aging [20]. Consequently, it can be surmised that the degree of intramuscular fatty infiltration in older individuals lowers physical performance [21].

Patients with lumbar degenerative spondylolisthesis showed multifidus muscle atrophy with erector spinae hypertrophy [22]. The multifidi actively stabilize the lumbar spine [23]. Atrophy or decreased function of these muscles could decrease lumbar spine stability. Therefore, in clinical practice, proper evaluation of the back muscles is essential to improve the stability of the spine. This study showed that aging, particularly sarcopenia, profoundly affects muscle degeneration and fatty infiltration of the lumbar spine. Therefore, generalized muscle atrophy can also influence fatty infiltration of the back muscles.

The prevalence of benign low back pain was shown to increase until the sixth decade of life and decrease thereafter [24]; however, the incidence of severe back pain continued to rise after the sixth decade. Elderly patients with chronic back pain for over 65 years had lower skeletal muscle mass than those without chronic back pain [25]. Body fat composition was higher in patients with chronic back pain. Therefore, fat infiltration of multifidi correlated with aging [26]. Our study also showed that the psoas index, a surrogate of generalized muscle atrophy, can profoundly influence back muscle atrophy and fatty infiltration beyond disc degeneration.

Exercise has been the treatment of choice for chronic back pain [27]. Resistance exercise has been shown to decrease intramuscular fatty infiltration in older individuals [28]. Therefore, considering that sarcopenia decreases back muscle mass and quality, exercise should be considered as a treatment strategy. Furthermore, physicians should pay attention to back muscles, as sarcopenia is associated with back pain.

Differences in total body mass between the sexes have been observed. Furthermore, trunk muscle mass showed a much larger difference between sexes than in other body regions [29]. Similarly, our data also showed that males had a larger back muscle CSA than females. Estrogen regulates muscular metabolism and maintains muscle mass [30,31]. However, sex hormones in females significantly decline during menopause. Thus, menopause induces remarkable functional and physiological changes in women, such as diminished muscle mass. The rapid decline in sex hormones has been shown to accelerate muscle atrophy in postmenopausal females [32]. Nonetheless, high-level physical activity in late perimenopausal females increased the appendicular lean muscle mass. Therefore, to decrease the risk of sarcopenia, physical activity and exercise should be actively considered in postmenopausal women with chronic back pain.

Our study has several limitations. First, we did not have any physical performance data of the subjects. Sarcopenia is defined as low muscular strength, muscle quantity, and physical performance. Therefore, it is necessary to evaluate the relationship between back muscles and physical performance. The second is the measurement of slice variability. Although we chose the bony landmark for measurement, the slice of the spine MRI was already manually adopted as an MRI scanner. Subsequently, we selected the optimal slice. However, all spine MRIs were conducted in a single medical center using the same MRI scan technique.

## 5. Conclusions

In conclusion, a systemic muscular decline is correlated with back muscle atrophy and fatty infiltration rather than disc degeneration. Therefore, a strategy for maintaining muscle mass is needed for older patients with chronic back pain.

## Figures and Tables

**Table 1:** Demographic data.

			Total	Male	Female	*p*-Value
Sex (male: female)			127	51	76	
Age			65.54 ± 14.93	61.41 ± 17.77	68.32 ± 12.03	0.01 *
Psoas CSA (cm^2^)			21.39 ± 8.19	28.61 ± 7.77	16.54 ± 3.63	0.00 *
Psoas index			8.13 ± 2.25	9.95 ± 2.15	6.90 ± 1.28	0.00 *
Back muscles (cm^2^)	L4–5	CSA (cm^2^)	43.77 ± 9.50	49.90 ± 9.32	39.65 ± 7.15	0.00 *
		Ratio of functional CSA	0.73 ± 0.12	0.78 ± 0.12	0.69 ± 0.11	0.00 *
	L5–S1	CSA (cm^2^)	33.57 ± 9.31	38.92 ± 9.70	29.98 ± 7.12	0.00 *
		Ratio of functional CSA	0.67 ± 0.13	0.73 ± 0.12	0.63 ± 0.12	0.00 *
Multifidus muscle (cm^2^)	L4–5	CSA (cm^2^)	16.14 ± 4.43	18.93 ± 4.71	14.27 ± 3.05	0.00 *
		Ratio of functional CSA	0.67 ± 0.16	0.74 ± 0.16	062 ± 0.15	0.00 *
	L5–S1	CSA (cm^2^)	18.04 ± 4.47	20.68 ± 4.26	16.27 ± 3.69	0.00 *
		Ratio of functional CSA	0.65 ± 0.16	0.72 ± 0.15	0.60 ± 0.16	0.00 *
	Pfirmann classification					
	L4–5	1 2 3 4 5	0 2 12 98 15	0 1 11 33 6	0 1 1 65 9	
	L5–S1	1 2 3 4 5	0 1 18 91 17	0 0 11 34 6	0 1 7 57 11	

CSA: cross-sectional area, * *p* < 0.05.

**Table 2:** Multivariant regression analysis of psoas index, age, Pfirrmann classification, and sex according to the ratio of functional CSA and CSA of back muscles.

		Ratio of Functional CSA		CSA of Back Muscles	
**Factors**		**L4–5**	**L5–S1**	**L4–5**	**L5–S1**
Psoas index	Mean ± SD (cm^2^)	8.13 ± 2.25			
	B	0.02	0.02	1.61	1.12
	SE	0.006	0.00	0.42	0.44
	β	0.49	0.49	0.38	0.27
	*p*	0.00 *	0.00 *	0.00 *	0.01 *
Age	Mean ± SD (cm^2^)	65.54 ± 14.93			
	B	−0.03	−0.00	−0.10	−0.10
	SE	0.00	0.00	0.05	0.05
	β	−0.30	−0.27	−0.16	−0.16
	*p*	0.00 *	0.00 *	0.06	0.07
Pfirmann classification	B	−0.02	−0.01	−1.92	−2.68
	SE	0.01	0.01	1.40	1.48
	β	−0.08	−0.06	−0.10	−0.15
	*p*	0.28	0.38	0.17	0.07
Sex	B	0.01	0.00	−4.22	−4.21
	SE	0.02	0.02	1.74	1.84
	β	0.07	0.03	−0.21	−0.22
	*p*	0.43	0.71	0.01 *	0.02 *
	Adj R2	0.47	0.44	0.42	0.35
	*p*	0.00 *	0.00 *	0.00 *	0.00 *

CSA: cross-sectional area, * *p* < 0.05.

**Table 3:** Multivariant regression analysis of psoas index, age, Pfirrmann classification, and sex according to the ratio of functional CSA and CSA of multifidus muscle.

		Ratio of Functional CSA		CSA of Multifidus	
**Factors**		**L4–5**	**L5–S1**	**L4–5**	**L5–S1**
Psoas index	Mean ± SD (cm^2^)	8.13 ± 2.25			
	B	0.02	0.03	75.34	92.33
	SE	0.00	0.00	18.26	20.19
	β	0.38	0.40	0.36	0.40
	*p*	0.00 *	0.00 *	0.00 *	0.00 *
Age	Mean ± SD (cm^2^)	65.54 ± 14.93			
	B	−0.00	−0.00	−10.26	−10.93
	SE	0.00	0.00	2.36	2.41
	β	−0.40	−0.33	−0.33	−0.31
	*p*	0.00 *	0.00 *	0.00 *	0.00 *
Pfirmann classification	B	−0.01	−0.04	−36.59	−132.51
	SE	0.02	0.02	60.95	57.65
	β	−0.03	−0.13	−0.04	−0.14
	*p*	0.61	0.05	0.54	0.02 *
Sex	B	−0.00	−0.00	−208.20	−151.88
	SE	0.03	0.03	75.94	83.63
	β	−0.01	−0.00	−0.22	−0.14
	*p*	0.89	0.97	0.00 *	0.02 *
	Adj R2	0.49	0.46	0.55	0.57
	*p*	0.00 *	0.00 *	0.00 *	0.00 *

CSA: cross-sectional area, * *p* < 0.05.

## Data Availability

The datasets used and/or analyzed during the current study are available from the corresponding author upon reasonable request.
